# The Effect of Antineoplastic Drugs in a Male Spontaneous Mammary Tumor Model

**DOI:** 10.1371/journal.pone.0064866

**Published:** 2013-06-03

**Authors:** Stephanie N. Shishido, Emma B. Faulkner, Amanda Beck, Thu A. Nguyen

**Affiliations:** Department of Diagnostic Medicine and Pathobiology, College of Veterinary Medicine, Kansas State University, Manhattan, Kansas, United States of America; Faculté de médecine de Nantes, France

## Abstract

Male breast cancer is a rare disease. The limited number of clinical cases has led to the primary treatments for men being derived from female breast cancer studies. Here the transgenic strain FVB/N-Tg(MMTV-PyVT)634Mul/J (also known as PyVT) was used as a model system for measuring tumor burden and drug sensitivity of the antineoplastic drugs tamoxifen, cisplatin, and paclitaxel on tumorigenesis at an early stage of mammary carcinoma development in a male mouse model. Cisplatin treatment significantly reduced tumor volume, while paclitaxel and tamoxifen did not attenuate tumor growth. Cisplatin treatment was shown to induce apoptosis, grossly observed by reduced tumor formation, through reduced Bcl-2 and survivin protein expression levels with an increase in caspase 3 expression compared to control tumors. Tamoxifen treatment significantly altered the hormone receptor expression levels of the tumor, while additionally upregulating Bcl-2 and Cyclin D1. This suggests an importance in hormonal signaling in male breast cancer pathogenesis. The results of this study provide valuable information toward the better understanding of male breast cancer and may help guide treatment decisions.

## Introduction

Male breast cancer accounts for 1% of all breast cancer cases in the United States, while it causes approximately 0.5% of all male cancer deaths [1]. Knowledge of this malignancy and appropriate therapies remain limited due to rarity of large cohorts of male breast cancer patients. Treatment for male breast cancer is therefore extrapolated from controlled clinical studies conducted in women [2]. Male breast cancer most commonly presents as late stage painless, firm masses in the subareolar location that become fixed to the pectoralis major muscle and the skin [3]. Male breast cancer is diagnosed in later stages than female breast cancer, leading to a tendency of small neoplasms to spread to the axillary lymph nodes. The 5-year survival rate for metastatic breast cancer in male patients is less than 20%, while the median survival is only about 15 months [4], [5]. Prognosis of male breast cancer is similar to stage matched females.

Male breast cancer differs from female breast cancer in many aspects. Most notably male breast cancer is diagnosed at older ages, presents at higher stage, has a bimodal age-frequency [4], [6], racial differences [6], distinct histological subtypes, immunophenotypic variations [4], [6], [7], low survival rates [4], and differential genetic mutations, such as CYP17 polymorphism [8], androgen receptor (AR) [9], and CHEK2 mutations [10]. Male breast cancer has also been shown to have a higher frequency of hormone receptor (HR), estrogen receptor (ER) and progesterone receptor (PR), expression (80–90%) compared to females (75%) [4], [5]. It is unclear if there is a relationship between ER+ male breast carcinomas and patient survival [4], [11], [12]. This may be due to differences in ER function in males as compared to females [13]. There is an up-regulation of ER expression in males due to the naturally lower estrogen levels in the tissue microenvironment, leading to an increase in estrogen targets [14]. An example of this is Bcl-2, which is an inhibitor of apoptosis, and has also been found to be overexpressed in male breast cancer [15].

The molecular subtypes of male breast cancers are based on the expression of certain protein markers in the neoplastic tissue, which are used to evaluate their association with the observed pathological features and patient outcome. It is important to note that the HR positivity of male breast carcinomas may not have the same prognostic value as female breast cancer. It is unclear whether the human epidermal growth factor receptor 2 (HER2) plays a prognostic or predictive role in male breast cancer [16], [17]. In a comparative study between male and female invasive breast carcinomas, the most common phenotype was luminal A (HR+/HER2−), while HR− and HER2+ were not identified in male patients [18]. In another study luminal A tumors were 82.8%, luminal B (HR−/HER2+) tumors found in 6.2%, and basal-like (HR−/HER2−) tumors were found in 9.6% of the male breast cancer cohort [19]. Contrary to these, another series reported to have no significant difference between tumor subtypes [20]. These studies show that the distribution of molecular subtypes in male breast cancer varies, but that it is also different compared to the female breast cancer cohorts. This is indicative of a pathological difference in carcinogenesis between males and females.

Certain populations have a higher risk of developing male breast cancer. The major risks are either genetic factors or hormone imbalance. Approximately 20% of males with breast cancer have a family history of breast or ovarian cancer [21]. Mutations in the *BRCA1* or *BRCA2* genes are the strongest known genetic risk factors for male breast cancer. More specifically the *BRCA2* gene mutation has a 7% lifetime risk of male breast cancer [22], which is a greater risk than females with a genetic predisposition of this disease. A change in the ratio of estrogen and testosterone is also an important factor contributing to male breast cancer. Individuals with Klinefelter’s syndrome have low testosterone levels, increasing the lifetime risk of developing male breast cancer to approximately 5% [21].

The commonly used therapeutic approach involves mastectomy with axillary lymph node evaluation and hormonal therapy, with potentially additional adjuvant chemotherapy. Hormonal therapy, primarily tamoxifen, is the mainstay of treatment for male breast cancer and is considered to be the first line treatment for metastatic male breast cancer, with an overall response rate of 49% [23]. In another study [24], it was reported that tamoxifen increased the 5-year actuarial survival (61% verse 44%) and disease free survival (55% verse 28%) rates of 39 male breast cancer patients compared with the historical control group [25]. Men in general tolerate tamoxifen well, with the most common side effects reported as decreased libido, weight gain, hot flashes, mood alteration and depression. However, it is important to note that there have been no randomized trials to evaluate the real response rates or the toxicity of tamoxifen in men. The role of other antineoplastic drugs commonly used for female breast cancer treatment has yet to be determined in male patients.

Advancements in diagnosis and treatment of female breast cancer have resulted in a steady decline in incidence and clinical outcome, while male breast cancer has been on a steady rise in incidence over the past several decades and a much slower improvement on clinical outcome [26]. The treatment for male breast cancer is extrapolated from female clinical trials, despite distinct clinical and pathological differences, as well as a lack of clinical improvement in patient outcome. There is a need for new clinical management of male breast cancer. The major hormonal differences between male and female patients regarding endocrinology and the breast carcinomas’ response, suggests a need to explore alternate treatment options.

The transgenic mouse strain FVB/N-Tg(MMTV-PyVT)634Mul/J (referred to as PyVT) is a novel *in vivo* model of mammary tumor formation and metastasis. The PyVT model has mammary tissue-specific expression of the Polyoma Virus middle T antigen driven by the mouse mammary tumor virus promoter [27]. PyVT premalignant tumors are morphologically heterogeneous with abnormal microvasculature, highly proliferative with atypical nuclei, and remain confined within the basement membrane prior to lung metastasis [28]. Studies show that even at early stages of mammary development the mammary fat pads were clearly abnormal with irregular growth of side branches, enlarged terminal buds, and large tumorous masses. Male animals generally develop mammary tumors by 15 weeks of age and reach maximum tumor burden around 25 weeks of age.

This study focuses on utilizing the *in situ* generation of male mammary tumors by the PyVT model to determine the efficacy of antineoplastic drugs, cisplatin, paclitaxel, and tamoxifen in attenuating tumor growth. The potential benefits of each treatment option are revealed for a male mammary tumor model.

## Materials and Methods

### Ethics Statement

Husbandry of animals is conducted by the Comparative Medical Group (CMG) at the College of Veterinary Medicine at Kansas State University. The CMG animal facilities are fully accredited by the Association for Assessment and Accreditation of Laboratory Animal Care, International (AAALAC). The compliance to aspects of animal welfare law is regularly monitored by the veterinary staff. Animal care and use protocols were approved by the Institutional Animal Care and Use Committee (IACUC) at Kansas State University (Protocol Number: 2975), Manhattan following NIH guidelines.

### Mouse Model

PyVT mice were purchased from the Jackson Laboratory (Bar Harbor, Maine). The mice were monitored every other day to check for the appearance of tumors. The tumor size was measured in two dimensions with calipers. Mice were observed for abnormal behavior, appearance or weight loss. If the animals showed any signs of pain, extreme tumor growth (greater than 1.5 cm) or loss of body condition, they were humanely euthanized before the end of the experimentation period. At different time points (10, 15, 20 weeks of age) the mice were sacrificed to examine mammary epithelium and tumor formation. All treatments were conducted on mice determined to be in an early tumor development stage, which was 15 weeks of age. Average weight of this age group was 28–32 grams. Mice were randomly divided into four experimental groups: (1) control animals given the vehicle solvent (DMSO); (2) animals treated with 3.5 mg/kg cisplatin; (3) animals treated with 10 mg/kg paclitaxel; and (4) animals treated with 40 mg/kg tamoxifen. All treatments were administered as an intraperitoneal injection.

### Antibodies

Primary antibodies to Survivin (sc-8807, goat polyclonal), Caspase 3 (sc-56046, mouse monoclonal), Cyclin D1 (sc-8396, mouse monoclonal), Bcl-2 (sc-492, rabbit polyclonal), ERα (sc-8002, mouse monoclonal), ERβ (sc-8974, rabbit polyclonal) and PR (sc-166170, mouse monoclonal) from Santa Cruz Biotechnology (Santa Cruz, CA); anti-GAPDH (2118, rabbit monoclonal) and anti-HER2 (4290, rabbit monoclonal) from Cell Signaling (Boston, MA) were used for both western blot and immunohistochemistry (IHC).

### Immunohistochemistry

All tumors were fixed in a solution of 10% formaldehyde and embedded into paraffin prior to sectioning them onto slides at a 5 µm thickness. Paraffin sections (5 µm) were dried at 60°C for 25 minutes. Deparaffinization was performed with 100% xylene and 100%, 90%, 75%, 50% ethanol. Antigen retrieval was performed in 1× Citrate buffer solution and steam. Slides were then incubated overnight at room temperature with a polyclonal antibody (1∶50 dilution). After washes in PBS, slides were successively incubated with biotinylated secondary antibodies (1∶1000) for 15 minutes. Slides were washed and immunostains were amplified by incubation with Avidin Biotin Complex (ABC) for 10 minutes accordingly. Cells were visualized with 3,3-diaminobenzidine (DAB) followed by a hematoxylin counterstain. The sections were viewed and the images captured with a Nikon 80i microscope.

### Western Blot Analysis

Mammary gland tumor tissue were homogenized in 500 mL of lysis buffer (20 mM Tris pH 7.5, 0.5 mM EDTA, 0.5 mM EGTA, 0.5% Triton X-100) at 1∶1000 dilution of protease inhibitors (Sigma-Aldrich, Saint Louis, MO). Tissue was homogenized using the OMNI Bead Ruptor 24 at a speed of 5.65 m/s for 45 seconds, followed by centrifugation at 13,000 rpm for 30 minutes at 4°C. Twenty-five micrograms of whole-cell extract was resolved by 10% SDS polyacrylamide gel electrophoresis (PAGE) and transferred to nitrocellulose membrane (Midwest Scientific, Saint Louis, MO). Nitrocellulose membranes were blocked with 0.5% milk in Tris-Buffered Saline and Tween 20 (TBST) using a SNAP i.d. device (Millipore) at room temperature. Membranes were then incubated with primary antibody at a 1∶1000 dilution, followed by HRP-linked secondary antibodies (1∶2000). Western blots were detected by enhanced chemiluminescence detection reagents (Pierce, Rockford, IL) and visualized by Fluorochem E imaging system (ProteinSimple, Santa Clara, CA).

### Statistical Analysis

Data is presented as the mean ± the 95% confidence interval of a minimum of three samples for molecular analysis and six samples for animal studies. Significance was determined at a P-value ≤0.05.

## Results

### Characterization of the PyVT Mouse Model and Effects of Treatment on Hormone Receptor Expression

FVB/N-Tg(MMTV-PyVT)634Mul/J male transgenic mice developed tumors as early as 14 weeks of age. All 10 mammary pads developed tumors with the maximum tumor burden achieved around 25 weeks of age. Tumor development in this spontaneous model was divided into 3 stages based on the extent of tumor size, the frequency of tumor formation, and whether it has metastasized to the lungs. Pre stage of PyVT tumor development occurred from 10–13 weeks of age and consisted of a pre-cancerous condition where no tumors were palpated and the mammary tissue appeared normal on gross observation. The Early stage of development represents solid tumor formation within the breast tissue at 15–18 weeks of age. This stage consisted of the gross observation of 1–2 solid tumors. The Late stage consisted of the presence of all 10 primary mammary tumors and secondary lung metastasis, which occurred after 20 weeks of age. The presence of metastases was confirmed by hematoxylin and eosin (H&E) staining of representative sections of the lung and histopathological review. This report focused on the Early stage of tumor formation and examined the effect of antineoplastic drugs on tumor growth at this stage.

**Figure 1:**
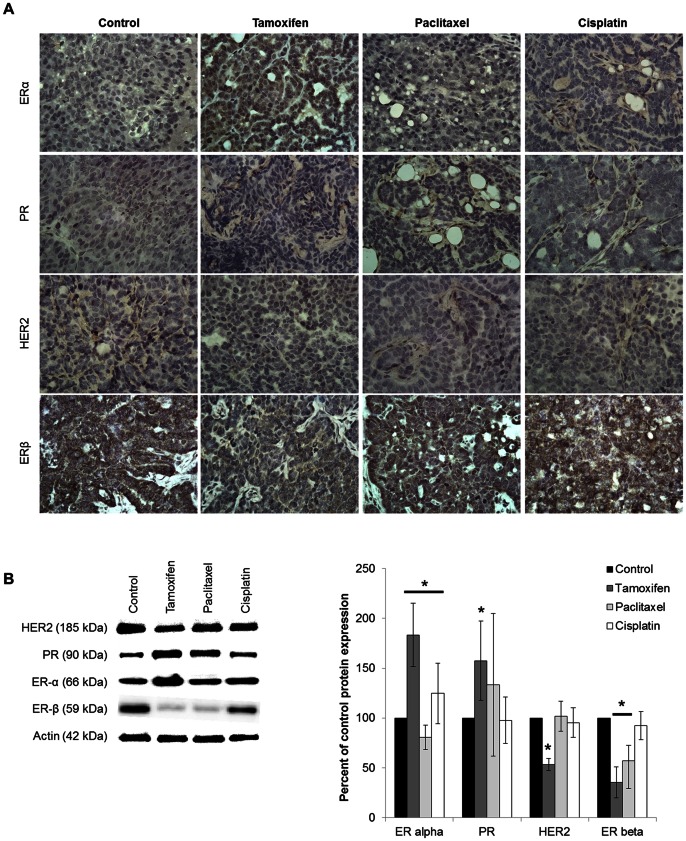
Male PyVT phenotype during early tumor development. A) Immunohistochemisty of tumor phenotype from PyVT males during tumor development. Paraffin-embedded sections stained with antibodies against estrogen receptor (ERα and β), progesterone receptor (PR), and human epidermal growth factor receptor 2 (HER-2) at the early stage of development. Proteins staining: brown, counterstaining: blue (hematoxylin). Images represent only 1 of 6 animals per group at a 60X magnification. B) Representative Western blot (n = 1) for hormone receptor expression in tumors isolated from male PyVT. Graphical representation shows the percent of control protein expression determined by pixel intensity of ERα, PR, HER2, and ERβ in PyVT male tumors treated with DMSO (control), tamoxifen (40 mg/kg), paclitaxel (10 mg/kg) or cisplatin (3.5 mg/kg) via 7 IPs during early tumor development. n = 4. *P-value <0.05 compared to control.

The mammary tumors were isolated from each treatment group to determine hormone receptor (ER, PR, and HER2) expression. Immunohistochemistry of the control tumor sections show weak positive staining of HER2 and strong positive staining of ERβ (Fig. 1A). Tamoxifen treatment resulted in an increase in positive nuclear staining of ERα in the tumors isolated, while decreasing the positive staining of ERβ. Western blot analysis was conducted to determine quantifiable expression levels of each hormone receptor in treated tissue. Controls tumors were shown to express ERα, ERβ, PR, and HER2 (Fig. 1B). Tamoxifen treated animals had an increased expression of ERα (P-value = 0.0021) and PR (P-value = 0.0300; Fig. 1B), while inducing a significant decrease in HER2 (P-value = 0.0002) and ERβ expression (P-value = 0.0002). Mice receiving paclitaxel treatment had a significant reduction in ERα and ERβ expression compared to control mice (P-value = 0.0201 and 0.0219, respectively), with no change in PR and HER2 expression. Interestingly animals treated with cisplatin showed no change in ERα, ERβ, PR, or HER2 expression, suggesting that treatment does not affect expression of the molecular markers.

### Effect of Cisplatin on Early Development of PyVT Mice

**Figure 2:**
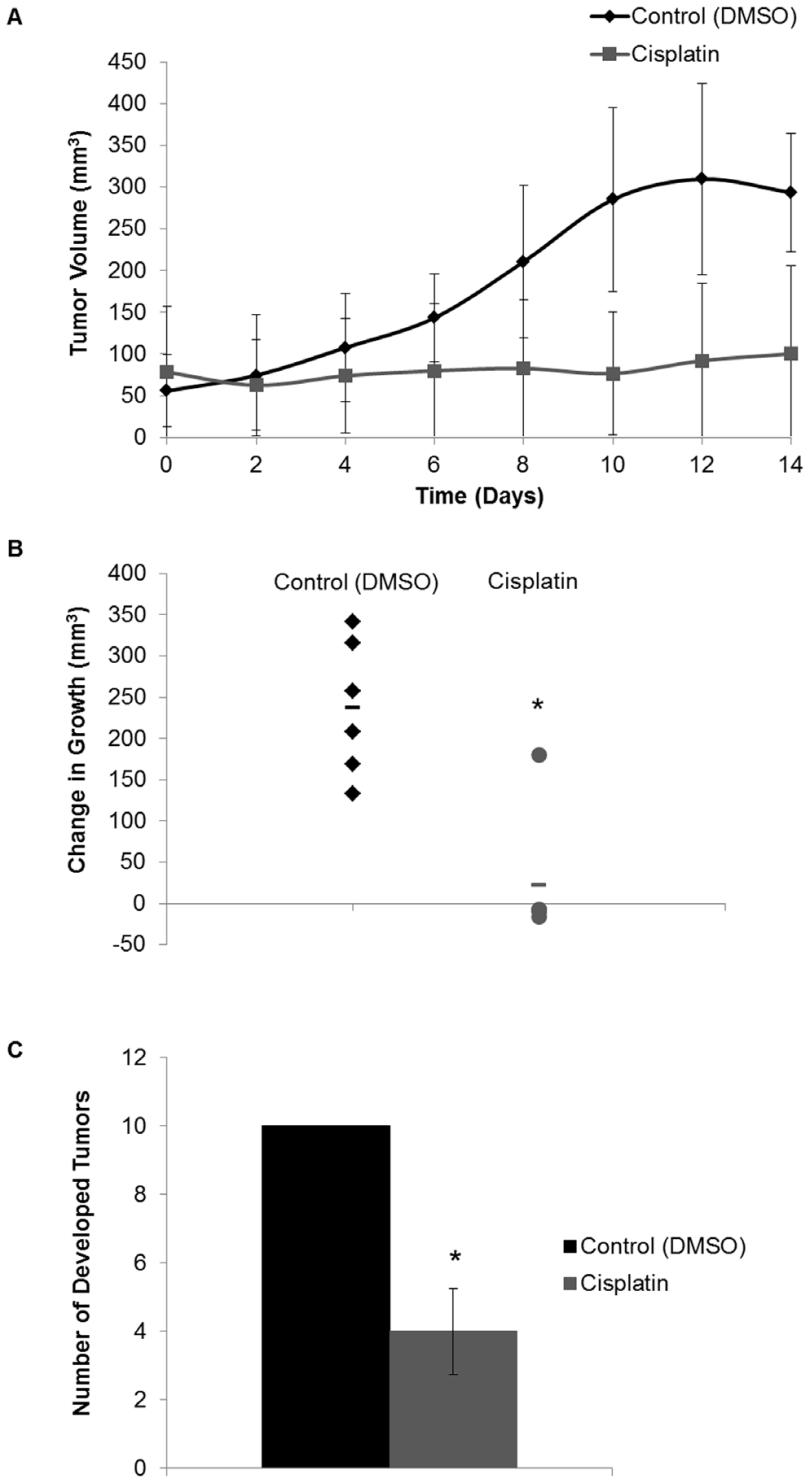
Tumor growth (mm^3^) in PyVT male mice treated with cisplatin. Tumors measured in two dimensions with calipers every 2 days prior to administration of treatment during early tumor development. A) The tumor size is expressed over the 14 day treatment period for the DMSO (control) and cisplatin (3.5 mg/kg) treated PyVT mice. Days 0–12 represent the days of the 7 IP injections, day 14 represents the end of the study with measurements prior to tissue harvest. B) The overall change in tumor size after treatment with DMSO (control) or cisplatin (3.5 mg/kg) via 7 IP injections. C) Number of developed tumors per PyVT male mouse during development. Tumors identified grossly during the early stage of tumor development after a 14 day period with either treatment with DMSO (control) or cisplatin (3.5 mg/kg) via 7 IP injections. *P-value <0.05 compared to control.

Tumor growth over a 14 day period with cisplatin treatment every other day indicates a significant effect of treatment on neoplastic development during the Early stage of tumor formation (Fig. 2). The initial tumor volume for all mice was 66.86±21.99 mm^3^. There was a significant difference in tumor volumes between DMSO and cisplatin treated mice from day 8 to day 14 (Fig. 2A). The final tumor volume for the control DMSO treated mice was 293.33±71.39 mm^3^, while the cisplatin treated mice had a final volume of 100.18±105.78 mm^3^. The change in tumor volume over the 14 day period shows a significant reduction of 215.59 mm^3^ with cisplatin treatment compared to control (P-value = 0.00044; Fig. 2B). This is a 90.71% difference between the overall changes in tumor growth after treatment with cisplatin.

Control mice have a total of 10 mammary fat pads that developed tumors during the treatment period. Treatment with cisplatin significantly reduced the number of tumors developed compared to the control group (P-value <0.0001; Fig. 2C). A total of 4 tumors developed with cisplatin treatment during the 14 day period.

### Effect of Paclitaxel on Early Development of PyVT Mice

**Figure 3:**
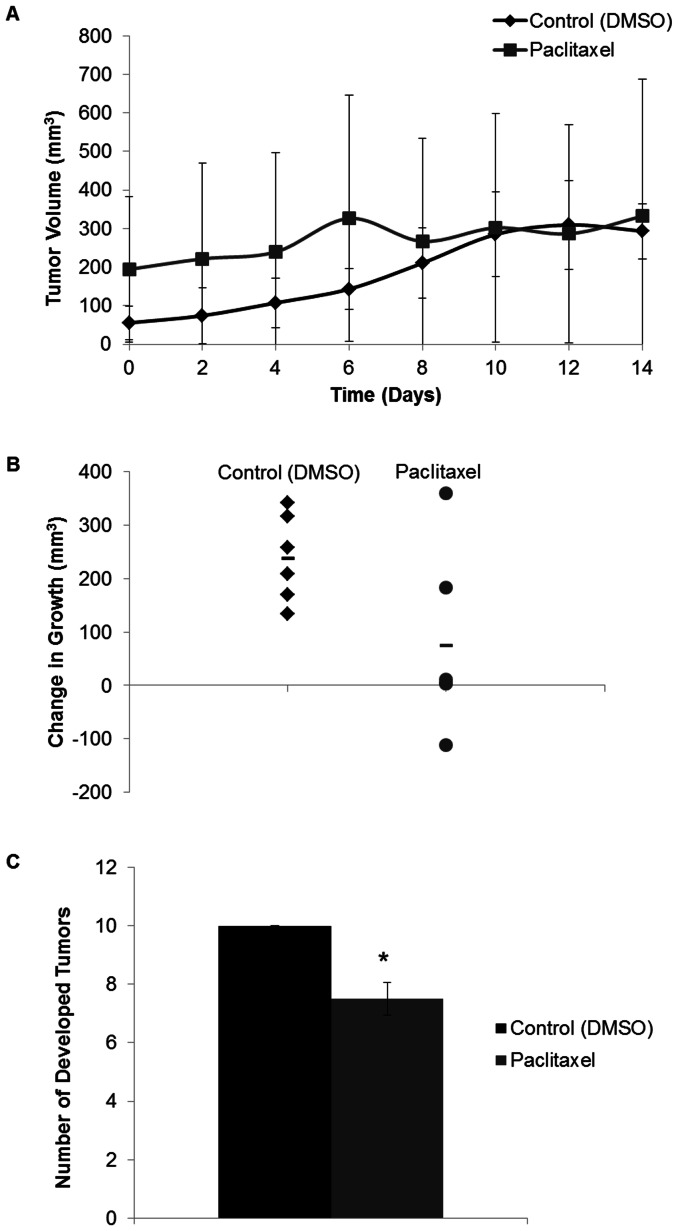
Tumor growth (mm^3^) in PyVT male mice treated with paclitaxel. Tumors measured in two dimensions with calipers every 2 days prior to administration of treatment during early tumor development. A) The tumor size is expressed over the 14 day treatment period for the DMSO (control) and paclitaxel (10 mg/kg) treated PyVT mice. Days 0–12 represent the days of the 7 IP injections, day 14 represents the end of the study with measurements prior to tissue harvest. B) The overall change in tumor size after treatment with DMSO (control) or paclitaxel (10 mg/kg) via 7 IP injections. C) Number of developed tumors per PyVT male mouse during development. Tumors identified grossly during the early stage of tumor development after a 14 day period with either treatment with DMSO (control) or paclitaxel (10 mg/kg) via 7 IP injections. *P-value <0.05 compared to control.

The initial tumor volume for all mice treated with either paclitaxel or DMSO was 137.34±93.05 mm^3^. There was not a significant difference in tumor volumes between treatment groups at any time during the 14 day treatment period (Fig. 3A). The change in tumor growth over the 14 day treatment period indicated that paclitaxel significantly attenuated tumor growth (P-value = 0.029, Fig. 3B). The control mice had an overall tumor growth of 237.68±65.78 mm^3^, while those mice treated with paclitaxel grew by 75.10±134.57 mm^3^. Additionally, paclitaxel treatment significantly reduced the tumor burden by an average of 2.5 tumors (P-value = 0.00022, Fig. 3C).

### Effect of Tamoxifen on Early Development of PyVT Mice

**Figure 4:**
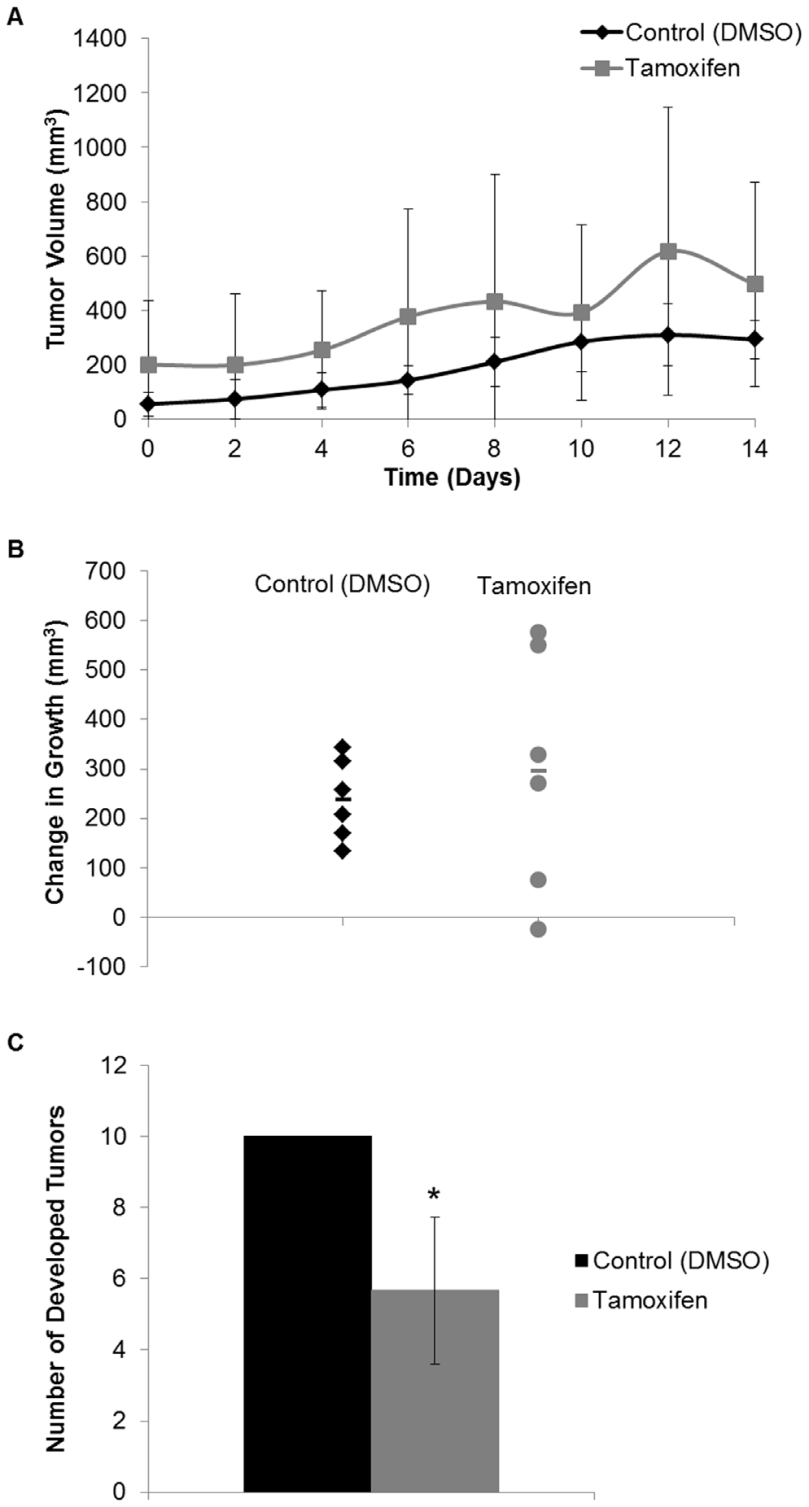
Tumor growth (mm^3^) in PyVT male mice treated with tamoxifen. Tumors measured in two dimensions with calipers every 2 days prior to administration of treatment during early tumor development. A) The tumor size is expressed over the 14 day treatment period for the DMSO (control) and tamoxifen (40 mg/kg) treated PyVT mice. Days 0–12 represent the days of the 7 IP injections, day 14 represents the end of the study with measurements prior to tissue harvest. B) The overall change in tumor size after treatment with DMSO (control) or tamoxifen (40 mg/kg) via 7 IP injections. C) Number of developed tumors per PyVT male mouse during development. Tumors identified grossly during the early stage of tumor development after a 14 day period with either treatment with DMSO (control) or tamoxifen (40 mg/kg) via 7 IP injections. *P-value <0.05 compared to control.

The tamoxifen treated mice and respective control mice began treatment with an initial tumor volume of was 127.91±122.53 mm^3^. Tamoxifen treatment did not affect tumor growth compared to the control animals during the treatment period (Fig. 4A and 4B). There was, however, a significant reduction in tumor burden by approximately 4 tumors (P-value = 0.00478, Fig. 4C).

### Protein Expression in Isolated PyVT Tumors

**Figure 5:**
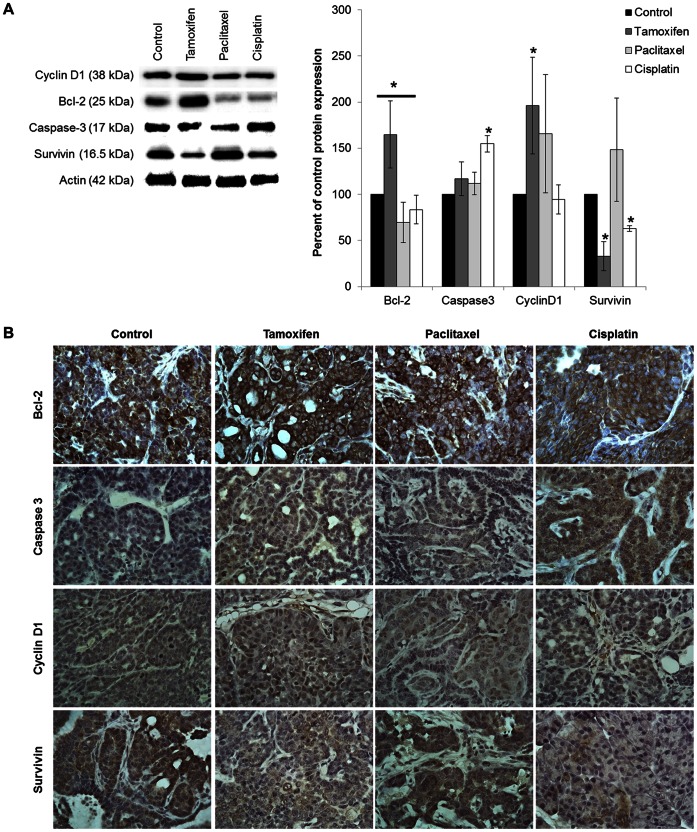
Expression of molecular markers Bcl-2, caspase 3, cyclin D1, and survivin. Tumors isolated from PyVT males treated with DMSO (control), tamoxifen (20 mg/kg), paclitaxel (10 mg/kg), or cisplatin (3.5 mg/kg). A) Representative Western blot (n = 1) of protein expression in tumors isolated from male PyVT. Graphical representation shows the percent of control protein expression determined by pixel intensity of Bcl-2, caspase 3, cyclin D1, and survivin in PyVT male tumors during early tumor development. n = 4. *P-value <0.05 compared to control. B) Immunohistochemisty of tumors from PyVT males. Paraffin-embedded sections stained with antibodies against Bcl-2, caspase 3, cyclin D1, or survivin from PyVT males. Proteins staining: brown, counterstaining: blue (hematoxylin). Images represent only 1 of 3 samples per group at a 60X magnification.

Immunoblot analysis was conducted to determine the expression of molecular markers for male breast cancer, including Bcl-2, caspase-3, survivin, and cyclin D1 (Fig. 5A). Bcl-2 has been shown to correlate with low mitotic cell count and lower grade tumors, suggesting it can be an important biomarker in male breast cancer pathogenesis [29]. Tamoxifen increased Bcl-2 by 65% compared to control (P-value = 0.0131). Paclitaxel significantly reduced the expression of Bcl-2 by 22% (P-value = 0.0346). There was an insignificant decrease of 17% in Bcl-2 expression with cisplatin treatment. Immunohistochemistry indicates strong positive staining of Bcl-2 in control and treated tumors (Fig. 5B). Tamoxifen treated tumors appear to have a stronger staining of Bcl-2, confirming western blot analysis.

The ability to induce apoptotic signaling in the tumor cells was determined by analysis of caspase expression. Cisplatin increased caspase 3 expression by 55% (P-value <0.0001) compared to control tumors (Fig. 5A). Tamoxifen and paclitaxel treatment did not change the expression of caspase 3. Control tumors have very weak positive staining for caspase 3. All antineoplastic compounds show an increase in positive staining compared to control, but cisplatin and tamoxifen have the strongest positive staining for caspase 3 (Fig. 5B).

Tumor cells are highly proliferative; therefore we explored the expression of cyclin D1 as a biomarker for cell proliferation. Cyclin D1 is a key cell cycle regulator in which over expression results in rapid progression from G1 to S phase in mitosis [30]. Analysis of cyclin D1 expression indicated that tamoxifen significantly increased expression by 96% (P-value = 0.0115), while paclitaxel and cisplatin did not significantly alter expression levels (Fig. 5A). Control tumors had weak positive staining for cyclin D1. Tamoxifen treated tumors had a strong positive staining, while paclitaxel and cisplatin treated tumors had weak positive staining for cyclin D1 (Fig. 5B).

Proteins that inhibit apoptosis provide protection for tumor cells against cytotoxic compounds. Survivin is a member of the inhibitors of apoptosis protein family that is expressed during embryogenesis and in tumor cells as an anti-apoptotic protein that is capable of regulating mitosis [31], [32], [33]. Survivin is highly expressed in a range of tumors and its expression correlates with both accelerated relapse and chemotherapy resistance [34]. Tamoxifen and cisplatin treatment significantly reduced survivin expression by 77% (P-value = 0.0019) and 48% (P-value <0.0001), respectively (Fig. 5A). Immunohistochemistry of the control tumors showed strong positive staining for survivin (Fig. 5B). Tumors treated with tamoxifen and cisplatin had weak positive staining for survivin compared to paclitaxel and control tissue.

### Pathological Review of Male Mammary Tumors

Pathological review of the mammary tumors was conducted for each treatment group. The tumors were categorized as either early carcinoma or late carcinoma. An early carcinoma consisted of a moderately demarcated neoplasm with closely packed nests and acini of proliferative neoplastic epithelial cells with cellular atypia and invasion of the basement membrane. The late carcinoma featured poorly demarcated neoplasms composed of sheets of tightly packed nest/acini of neoplastic epithelial cells separated by fibrovascular stroma with a loss of mammary architecture, increased proliferation, and more extensive invasion.

Control tumors were characterized by focal hyperplasia with normal lymph nodes and adipose tissue or early carcinoma lesions. Treatment with cisplatin and paclitaxel had no significant change in the histopathology, and tumors remained characteristic of early carcinoma. Mice treated with tamoxifen developed tumors characteristic of late carcinoma, suggesting an increase in malignancy due to treatment.

## Discussion

The transgenic PyVT model was used here for translational studies of male breast cancer due to its clinically relevant pathology and protein expression profile. Models for male breast cancer are limited, but the lack of clinical patients make this transgenic mouse model vital to gain an understanding about the pathological differences in male breast cancer compared to the female counterparts. The male PyVT model has provided an opportunity to address the efficacy of treatment using the antineoplastic drugs: tamoxifen, paclitaxel, and cisplatin. Tamoxifen is better known as a selective estrogen receptor modulator (SERM) because of its multiple activities [35]. Due to the high hormone receptor positivity in male breast cancer, tamoxifen is the standard adjuvant therapy. Paclitaxel, approved by the Food and Drug Administration (FDA) to treat ovarian and breast cancer, is a first-line treatment of female metastatic breast cancer. Paclitaxel promotes the stable assembly of microtubules and inhibits their de-polymerization [36], therefore interfering with the normal function of microtubules and preventing the progression of the cell cycle [37]. Cisplatin is a platinum based chemotherapy drug used to treat a variety of cancers through the formation of platinum-DNA adducts that induce cell cycle arrest [38], [39]. These compounds have drastically different modes of action. Here we determined the efficacy of each in attenuating mammary tumor growth in a male model.

This study shows that early male mammary tumor formation is significantly attenuated by cisplatin treatment, while tamoxifen and paclitaxel have no effect on tumor growth. This suggests that treatment options need to be reconsidered for male breast cancer patients. Tamoxifen is the current primary treatment, but results indicate that it does not efficiently attenuate tumor growth. Cisplatin was shown to be the more efficient antineoplastic tested, suggesting a switch in compounds for primary treatments of male breast cancer patients. Interestingly all three antineoplastic compounds, cisplatin, paclitaxel, and tamoxifen reduced the total number of developed tumors, indicating they could have a chemopreventive property.

Hormone receptor expression is the primary way to profile mammary carcinomas. The male PyVT tumors were shown to be ERα/β+, PR+, and HER2+. Tamoxifen treatment increased the expression of both ERα and PR, while resulting in a decreased expression of HER2 and ERβ, indicating an inverse relationship between the ER isoforms due to tamoxifen treatment. Hormone sensitive tumors are typically based on the expression of only ERα. The role of ERβ in the pathology and treatment of breast cancer remains largely unknown. The functions of these two estrogen receptors are drastically different in response to both estrogen and anti-estrogenic compounds [40], [41]. Multiple reports show that estrogen exposure to ERα expressing breast cancer cells lead to an increase in proliferation, while exposure to ERβ expressing cells, either alone or in combination with ERα, results in inhibition of cellular proliferation [42], [43], [44]. This suggests that ERβ may function more as a tumor suppressor than a tumor promoter. The expression of ERβ has been found in 47% of breast tumors classified as ERα negative [45]. Interestingly paclitaxel also reduced the expression of ERβ, but did not affect expression of ERα, PR, or HER2. The clinical relevance of ERβ expression is uncertain, multiple studies indicate a correlation with improved survival [46], [47], while others suggest little correlation or worse prognosis [48], [49]. These findings emphasize the need to further elucidate the function of ERβ in the pathology and treatment of breast cancer.

Tumor regression occurs when the rate of cellular proliferation is less than the rate of cellular death. To determine the apoptotic signaling due to the antineoplastic treatments, caspase 3 expression levels were measures in all tumors. Cisplatin was the only compound to induce a significant increase in caspase 3, indicating induction of apoptosis due to treatment. This is observed grossly by a significant reduction in tumor size. Tamoxifen and paclitaxel did not have an observable apoptotic effect. The inhibitor of apoptosis, survivin, was measured in tumor tissue after treatment with the antineoplastic compounds. There was a significant decrease in the expression of survivin after tamoxifen or cisplatin treatment. This indicates that both treatments reduce the anti-apoptotic signals, thus promoting cellular death. Cisplatin treatment therefore promotes and induces apoptosis, resulting in a decreased tumor volume. Tamoxifen treatment only promotes apoptosis, thus sensitizing the cell for apoptotic signaling, but not directly leading to cellular death.

Bcl-2 is another regulator of apoptosis, but has been shown to be an important biomarker in male breast cancer pathogenesis, correlating with low mitotic cell count and smaller tumors with lower histological grade [29]. Bcl-2 is also a critical biomarker for female breast cancer in predicting patient survival [50]. In this study tamoxifen was shown to increase Bcl-2 expression, while paclitaxel and cisplatin decreased expression levels compared to control tumors. The expression of survivin with other anti-apoptosis genes like Bcl-2 reduces apoptosis of cancer cells [51]. Bcl-2 expression is expected to correlate with survivin, which was observed with cisplatin treatment in which both proteins have a reduced expression compared to control tumors. Interestingly tamoxifen treated tumors show an inverse relationship between Bcl-2 and survivin. Bcl-2 proteins are found as dimers in the outer mitochondrial membrane [52]. The physiological role of Bcl-2 expression and control of homeostasis in normal breast tissue is suggested to involve upregulation by estradiol and down-regulation by progesterone [53]. Additionally in breast cancer cells estradiol was shown to stimulate, while progesterone inhibited Bcl-2 protein expression [54]. This suggests Bcl-2 regulation through the hormone receptors, specifically the upregulation of Bcl-2 by ERα and down-regulation by PR. Here we show that tamoxifen treatment increases expression of ERα and decreases ERβ, which may lead to the increased expression of Bcl-2.

Highly proliferative neoplastic cells and high histological grade tumors have been associated with increased expression of cellular markers for proliferation. Abnormal cyclin D1 expression is common in female breast cancer [55], [56]. Prognostic relevance of the proliferative proteins cyclinD1 and Ki67 have not been confirmed in male breast cancer patients. CyclinD1 has been shown to be overexpressed in 77% of male breast cancer [29]. Interestingly, in a cohort of male breast cancer patients cyclin D1 overexpression was predicative of better patient survival, while high levels of cyclin A and B expression increase the risk for breast cancer related death by 2–3 fold [57]. In male PyVT mice, treatment with tamoxifen increased cyclin D1 expression without significantly altering tumor growth. This conflicts with the findings from Nilsson *et al.* while affirming that cyclin D1 may not be a suitable molecular marker for male breast cancer.

The differences in the physiology of female and male patients with breast cancer warrant a different treatment approach, specifically with regards to hormonal therapy. More research is needed to determine the role of anti-estrogenic compounds such as tamoxifen in male breast cancer treatment. Scattered reports are insufficient to recommend treatment guidelines. Based on the known differences in the biology of male and female breast cancer, it is only practical to consider treatment options that do not alter the hormonal signaling or at least not as a single treatment agent of breast cancer in men.

This study offers a unique opportunity to study the effects of certain antineoplastic drugs in a male mammary tumor model. The results show the effects of treatment with three accepted antineoplastic drugs that have not been effectively assessed in the male system due to low clinical occurrence rates of male breast cancer. The results of this study provide valuable information toward the better understanding of male breast cancer and may help guide treatment decisions.
